# Radiochemical analysis of the drain water sampled at the exhaust stack shared by Units 1 and 2 of the Fukushima Daiichi Nuclear Power Station

**DOI:** 10.1038/s41598-022-05924-2

**Published:** 2022-02-08

**Authors:** Asako Shimada, Yoshinori Taniguchi, Kazuo Kakiuchi, Saki Ohira, Yoshihisa Iida, Tomoyuki Sugiyama, Masaki Amaya, Yu Maruyama

**Affiliations:** Nuclear Safety Research Center, Japan Atomic Energy Agency, 2-4 Shirakata, Tokai-mura, Naka-gun, Ibaraki 319-1195 Japan

**Keywords:** Environmental sciences, Environmental impact

## Abstract

Radioactive gas of Unit 1 of the Fukushima Daiichi Nuclear Power Station was released from the exhaust stack shared by Units 1 and 2 through the venting line on March 12th, 2011. In the present study, radiochemical analysis of drain water sampled at the drain pit of the exhaust stack was conducted to study radionuclides released during venting of the Unit 1. Not only volatile ^129^I, ^134^Cs and ^137^Cs but also ^60^Co, ^90^Sr, ^125^Sb and Unit 1-originated stable Mo isotopes were detected. Although Unit 1-originated stable Mo isotopes were clearly detected, their amounts were quite low compared to Cs, suggesting that the formation of Cs_2_MoO_4_ was suppressed under the accident condition. Approximately 90% of iodine existed as I^−^ and 10% as IO_3_^−^ in November 2020. Furthermore, larger amount of ^129^I than ^137^Cs was observed, suggesting major chemical form of ^131^I was molecular iodine rather than CsI at the accident time. The ^134^Cs/^137^Cs radioactivity ratio decay-corrected to March 11th, 2011 was 0.86, supported the results that Unit 1 originated radiocesium in environment has smaller ^134^Cs/^137^Cs radioactivity ratio than Unit 2 and 3 originated radiocesium.

## Introduction

Because of the tsunami caused by The Great East Japan Earthquake, TEPCO’s Fukushima Daichi Nuclear Power Station (FDNPS) lost all electric power, followed by the severe accidents at Units 1, 2, and 3. The accident progression has been analyzed by many researches with emphasis on the thermal-hydraulics in the OECD/NEA projects such as Benchmark Study of the Accident at the Fukushima Daiichi Nuclear Power Station (BSAF)^1–8^ and Analysis of Information from Reactor Buildings and Contaminant Vessels of Fukushima Daiichi Nuclear Power Station (ARC-F). In addition, fission products (FPs) release, distribution of FPs in all over the plant, and source term to environment are also the topics treated in the projects. However, there are still many uncertainties such as a type of leakage, event timings, and chemical species of FPs due to lack of complete data to input calculation codes.

In order to reduce uncertainties concerning assessments of severe accidents, VERDON tests were performed at the CEA Cadarache Centre in which high burn-up UO_2_ and MOX fuels were heated in a furnace under various conditions to investigate FPs release and transportation^9–13^. The results indicated a rapid release kinetics for volatile (I, Cs, Te) and even for semi-volatile (Mo, Ba) FPs during oxidizing condition and re-volatilization of iodine during air injection on the contrary to Cs. Transportation of FPs are affected by not only atmosphere and temperature conditions but also chemical species. While many source term studies have assumed Cs chemical form as CsI and CsOH, recent studies pointed out possible formation of Cs_2_MoO_4_^5–7,14–18^, CsBO_2_^19^, Cs_2_CrO_4_^19,20^, Cs_2_Si_4_O_9_^19^, and CsAlSiO_4_^21^. The formation of these chemical species would be related to the leakage pathways since some reports indicated the production of Cs_2_Si_4_O_9_ and CsAlSiO_4_ via chemisorption of Cs onto stainless steel and reaction with concrete^22,23^. Furthermore, the production of Cs_2_Si_4_O_9_ and CsAlSiO_4_ is possibly related to the Cs-bearing microparticle found in environmental samples after the FDNPS accident^24–26^.

There are many reports related to the distribution of ^134,135,136,137^Cs, ^129,131^I, ^90^Sr, ^110m^Ag and ^132^Te in environment caused by the accident at the FDNPS^27–34^. The measured radionuclides concentrations were compared with those calculated by atmospheric transport and deposition model (ATDM) to estimate the atmospheric release of radionuclides during the FDNPS accident^27^. The detailed analysis of ^134^Cs/^137^Cs radioactivity ratio of the deposition indicated which reactor unit ultimately contaminated a specific area: while the contamination of most continental areas was caused by Units 2 and 3, the contamination of the northwest part near FDNPS was caused by Unit 1 because the ^134^Cs/^137^Cs radioactivity ratio was slightly lower than those in the other parts^27–29^. Furthermore, slight difference in the ^135^Cs/^137^Cs isotope ratio was reported for the area mainly contaminated by Unit 1^31^. Source term information is important to improve such analysis.

Recently, radiochemical analysis of samples collected inside of FDNPS buildings has been planned to improve severe accident analysis and source term. In the present study, drain water sampled at the drain pit of the exhaust stack shared by Units 1 and 2 was analyzed to obtain the information about components and chemical species of radionuclides released by vent of Unit 1. Cesium-134,137 and ^131^I are the important FPs of severe accident analysis because of their relatively high volatile and radioactivity. However, ^131^I was decayed out due to its short half-life (8.0 d). Therefore, ^129^I which has long half-life (1.57 × 10^7^ y) was measured to estimate the behavior of ^131^I.^32,33^ In addition, Mo isotopes and ^99^Tc, daughter nuclide of ^99^Mo, were analyzed because Mo was prone to be released as Cs_2_MoO_4_ when the reactor vessel was under oxidizing condition with sufficient water vapor. Furthermore, lower volatile FPs (^90^Sr and ^152,154^Eu) and actinide (Th, U, Pu, and Am) were analyzed to investigate the release of them as aerosols generated in reactor vessel and Molten Core Concrete Interaction.

## Materials and analysis method

### Drain water

**Figure 1:**
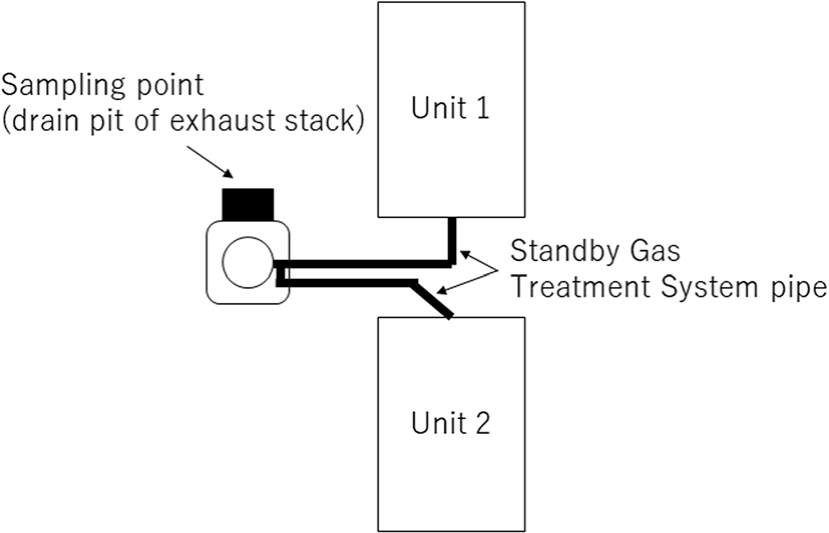
Diagrammatic illustration of the sampling point.

The sampling point was illustrated in Fig. 1. In the vent operation, the gas in the contaminant vessel was passed through the suppression chamber to reduce the radioactivity, and then, released into environment via stand by gas treatment system pipe and exhaust stack. The exhaust stack was shared by Units 1 and 2. It is considered that the vent of Unit 2 was failed since outflow side of the rupture disk in the vent line of Unit 2 was not contaminated, and the inside of the exhaust stack was mainly contaminated by vent of Unit 1. It is presumed that dew condensation water and rainwater in the stack were accumulated in the drain pit. Drain water was sampled in September 12, 2016. Salinity and pH of the drain water were preliminary measured using salinometer and pH test paper, and they were 0.01% and neutrality.

### Analysis of γ-emitting nuclides

γ-Ray spectra were measured by a Ge semiconductor detector (GC1020S-CJT-F-1/S-2002C, Mirion Technologies Canberra). Energy and efficiency of the γ-ray spectrum were calibrated using a mixed standard source of ^60^Co, ^133^Ba, and ^137^Cs, which was prepared as a filter paper (Amersham plc, UK). Therefore, the sample for quantitative analysis of drain water was prepared by transfusing 30 μl of sample solution into a filter paper to be the same sample form. The filter paper was doubly sealed in plastic bags. First, measurement of ^134^Cs and ^137^Cs was conducted on the drain water as it was. After that, the measurement of the nuclides excluding ^134^Cs and ^137^Cs, such as ^60^Co, ^106^Rh (daughter nuclide of ^106^Ru), ^125^Sb, and ^152^Eu, was conducted after Cs was eliminated using ammonium phosphomolybdate (AMP) because γ-rays from the other nuclides were considered to be hidden by the higher level of radioactivity of ^134^Cs and ^137^Cs in the drain water. The amount of AMP, HNO_3_ concentration and standing time were optimized to eliminate Cs and to remain the other elements before analysis of the drain water. Sample solutions for the optimization were prepared by diluting the standard solutions of B, Na, Mg, Al, Ca, Mn, Fe, Co, Ni, Se, Rb, Sr, Y, Zr, Mo, Ru, Rh, Ag, Sn, Sb, Te, Cs, Ba, Eu, Re (1 mg/ml solutions purchased from Fujifirm Wako Chemical CO.INC., Kanto Chemical CO.,INC., and Merck) and of Nb (0.1 mg/ml solution purchased from Accu Standard). Based on the optimization, 5 μl of standard solutions of Co, Se, Sr, Ru, Rh, Sb, Cs, Eu and Re (1 mg/ml) were added to 5 ml of drain water and HNO_3_ concentration was adjusted to 2 M. The drain water sample solution was stood for overnight. After the addition of 0.02 g of AMP to the drain water sample solution, it was stirred for 1 h. AMP was filtered using a syringe and a membrane filter unit of which pore size was 0.45 μm. The filtrate was assigned as Cs-eliminated drain water, and quantitative analysis of γ-ray emitting nuclides was conducted. Furthermore, γ-ray spectra of the Cs-eliminated drain water in a bottle (approximately 5.7 ml) were recorded for the qualitative analysis.

### Analysis of ^90^Sr

Five μl of Cs standard solution was added to the Cs-eliminated drain water, and then, the Cs elimination procedure using AMP was repeated one more time with the same manner of the first time. The obtained filtrate was assigned as the twice Cs-eliminated drain water. 0.1 ml of the twice Cs-eliminated drain water was taken into a bottle, and HNO_3_ and pure water were added to prepare 2 ml of 8 M HNO_3_ sample solution. This was assigned as DW-Sr sample.

0.5 ml of the Sr resin (Eichrom Technology, LLC) swollen with ultrapure water was packed into a column and conditioned by passing through 5 ml of 8 M HNO_3_. Next, the DW-Sr sample was loaded into the column. The bottle which had contained DW-Sr sample was rinsed with 0.5 ml of 8 M HNO_3_ 4 times and all rinse solutions were loaded into the column. After that, 2.5 ml of 8 M HNO_3_ was passed through the column 4 times. The extracted Sr was recovered by passing through 3 ml of 0.01 M HNO_3_. 0.2 ml of the recovery solution was sampled and mixed with 1.8 ml of 1 M HNO_3_ to measure Sr concentration with inductively coupled plasma mass spectrometer, ICP-MS, (Agilent 7700, Agilent Technologies Japan, Lts), and the recovery rate during chemical separation was evaluated. Ultima Gold LLT (PerkinElmer) was added to the remaining recovery solution, and β-ray spectrum was measured with liquid scintillation counter (PerkinRlmer Tri-Carb 3110TR) after reaching radioactive equilibrium between ^90^Sr and ^90^Y. Efficiency was corrected by a calibration curve obtained from the measurement of standard samples containing 20 Bq of ^90^Sr and ^90^Y, various concentration of HNO_3_ and Ultima Gold LLT.

### Analysis of ^99^Tc

Aliquot of the twice Cs-eliminated drain water was adjusted to approximately 5 ml of 1 M HNO_3_ solution (DW-Tc). 0.5 ml of the TEVA resin (Eichrom Technology, LLC) swollen with ultrapure water was packed into a column and conditioned with 1 M HNO_3_. The DW-Tc was loaded into the TEVA resin column. The bottle which had contained DW-Tc sample was rinsed with ultrapure water 3 times and all rinse solutions were loaded into the column. After that, 2.5 ml of ultrapure water was passed through the column 4 times. The extracted Tc was recovered by passing through 1.5 ml of 8 M HNO_3_. The recovery solution was appropriately diluted to measure Re with ICP-MS. The remaining recovery solution was also appropriately diluted and mixed with Ultima Gold LLT to measure β-ray of ^99^Tc with liquid scintillation counter (PerkinElmer Tri-Carb 3110 TR).

### Analysis of Mo isotopes

Molybdenum isotopes (mass number 92, 94, 95, 96, 97, 98 and 100) in a Mo standard solution (0.5 ng/mL, this solution was prepared by diluting a standard solution of 1 mg/ml of Mo, Fujifilm Wako Chemical Co. INC.) was measured using ICP-MS (Agilent 7700) and Sector field ICP-MS (Element 2, Thermo Fisher Scientific Co.) to study the effect of mass discrimination on the isotope ratios obtained by ICP-MS measurement. Reference values of the isotope ratio to ^98^Mo, *R*_true_, were divided by those of measured, *R*_meas_, and plotted against the mass difference of the isotope of interest from ^98^Mo, *Δ*m.

Previously, separation method for Mo using a TEVA resin was developed to purify Mo from rubble samples^35^. Because the dissolved solution of the rubble sample contained large amount of Fe^3+^ and Cr^6+^, which compete with Mo extraction on the TEVA resin, ascorbic acid was added to the sample solution to decrease their extraction by reducing to Fe^2+^ and Cr^3+^. In addition, HF was added to the sample solution to avoid precipitation of Nb. However, influence of Fe, Cr, and Nb in the drain water is negligible due to lower concentration of them. Therefore, sample solution of the drain water to load the TEVA resin was adjusted to 4 M HCl using ultrapure grade HCl solution (31%, Kanto Chemical Co.,INC.), and the solution was assigned as DW-Mo sample. 0.5 ml of the TEVA resin swollen with ultrapure water was packed into a column, followed by washing with 5 ml of 1 M HNO_3_, 3.4 ml of ultrapure water, and 2 ml of 4 M HCl. Then, 5 ml of the DW-Mo sample was loaded into the column. The bottle which had contained DW-Mo sample was rinsed with 0.5 ml of 4 M HCl 3 times and all rinse solutions were loaded into the column. Next, the column was rinsed with 3.4 ml of 4 M HCl and 0.5 ml of ultrapure water. Finally, extracted Mo was recovered by passing through 2.5 ml of 1 M HNO_3_. As an operation blank solution, 4 M HCl solution was prepared and the same separation operation was carried out. Recovered Mo isotopes were measured with sector field ICP-MS. Net count rates of Mo isotopes for drain water were obtained by subtracting the measured count rates of Mo isotopes for the operation blank solution from those for the drain water. Whereas, net count rates of Mo isotopes for Mo standard solution, 0.5 ng/ml, were obtained by subtracting the measured count rates for 1 M HNO_3_ solution from those for the Mo standard solution.

### Analysis of ^129^I^−^ and ^129^IO_3_^−^

It was reported that I^−^ was extracted on the solid extraction disk, Anion-SR (Empore disk, 3 M Co.), from 3 M NaOH solution, and I^−^ and IO_3_^−^ were extracted on the Anion-SR from 0.01 M HCl containing 0.1 M NaHSO_3_ because IO_3_^−^ was reduced to I^−^ by NaHSO_3_ in diluted HCl solution^36,37^. In the present study, 0.5 ml of drain water was adjusted to 5 ml of 2.5 M NaOH solution to analyze I^−^ and to 5 ml of 0.01 M HCl solution containing 0.01 M K_2_S_2_O_5_ to analyze the sum of I^−^ and IO_3_^−^. Instead of NaHSO_3_, K_2_S_2_O_5_ was used because NaHSO_3_ is unstable in a solid state, and formula number of Na_2_SO_3_, 126, is close to mass number of stable iodine, 127. As spike samples, a known amount (approximately 50 pg, “a” in the Eqs. (1) and (2)) of ^127^I^−^ or ^127^IO_3_^−^ was added to the drain water in the same solution conditions. In addition, 2.5 M NaOH solution and 0.01 M HCl solution containing 0.01 M K_2_S_2_O_5_ were also prepared as blank samples. The I^−^ or the sum of I^−^ and IO_3_^−^ were purified by Anion-SR using the reported method^36,37^. Briefly, Anion-SR was sequentially conditioned with 10 ml of acetone, 10 ml of methanol, 10 ml of pure water, 10 ml of 1 M HNO_3_ and 30 ml of pure water. Next, the sample solution was loaded to the Anion-SR, and then, it was rinsed with 5 ml of pure water 6 times. Finally, extracted I^−^ was recovered 9.5 ml of 1 M HNO_3_. A known amount of Cs was added into the purified I^−^ or (I^−^ + IO_3_^−^) solutions as an internal standard and a known amount of NaClO was added to uniform the chemical species to IO_3_^−^ and to stabilize iodine in 1 M HNO_3_ solution. The concentrations of ^127^I (“x” in Eq. (1)) and ^129^I (“y” in Eq. (2)) were measured by using ICP-MS (Agilent 7700). The amounts of ^127^I and ^129^I were calculated by using Eqs. (1) and (2), respectively.

1 $${\text{x}} = \left\{ {\left( {{\text{A}}/{\text{B}}} \right) \times {\text{a}}} \right\}/\left\{ {\left( {{\text{A}}^{\prime } /{\text{B}}^{\prime } } \right) - \left( {{\text{A}}/{\text{B}}} \right)} \right\}$$

2 $${\text{y}} = {\text{a}}/\left\{ {\left( {{\text{A}}^{\prime } /{\text{B}}^{\prime } } \right) - \left( {{\text{A}}/{\text{B}}} \right)} \right\}$$

where A and B were the measured amounts of ^127^I and ^129^I in a sample solution, respectively, A’ and B’ were the measured amounts of ^127^I and ^129^I in the spike sample solutions, respectively, and a was spiked amount of ^127^I. The amounts of x and y were obtained for chemical species of I^−^ and the sum of I^−^ and IO_3_^−^, and the amount of IO_3_^−^ was calculated by subtracting the amount of I^−^ from that of the sum of I^−^ and IO_3_^−^.

### Measurement of stable isotopes, Th and U in drain water

The drain water was diluted 200 times with 1 M HNO_3_ and the concentrations of ^59^Co, ^77,82^Se, ^88^Sr, ^99^Tc, ^101^Ru, ^103^Rh, ^121^Sb, ^133^Cs, ^153^Eu, ^185^Re, ^232^Th and ^238^U in the drain water were determined using ICP-MS (Agilent 7700) by calibration curve method.

### Measurement of Pu and Am

One (1) ml of the drain water was adjusted to 2 ml of 1 M HNO_3_ solution containing 0.1 M NaNO_2_ and assigned as DW-PuAm sample. Pulutonium-242- or ^243^Am-spiked drain water sample was prepared as the same manner and assigned as Pu-DW-PuAm and Am-DW-PuAm samples, respectively. 0.5 ml of TRU resin (Eichrom Technology, LLC) swollen with ultrapure water was packed into a column and conditioned with 1 M HNO_3_. Then, the sample solution was loaded into the column. The bottle which had contained the sample was rinsed with 1 M HNO_3_ 3 times and all rinse solutions were loaded to the column. The column was rinsed with 3.4 ml of 1 M HNO_3_. Extracted Am was recovered with 3.4 ml of 4 M HCl, and next, extracted Pu was recovered with 3.4 ml of 4 M HCl containing 0.02 M TiCl_3_. 0.3 ml of Sm solution (1 mg/ml) and 1 ml of HF were added to the recovery solutions of Am and Pu to precipitate AmF_3_ and PuF_3_ with SmF_3_. The micro precipitate was collected on PTFE membrane filter and dried with a little amount of ethanol. The filter was fixed with double-stick tape on a stainless-steel dish to subject to the measurement by α-ray spectrometer (α-Ray Detector BU-020-450-AS0, α-Ray Module SOLOIST,SEIKO EG&G ORTEC). The recovery rate of the micro precipitate was normalized with the recovery of ^147^Sm, which is a natural alpha-emitting radioisotope (half-life: 1.06 × 10^11^ y, alpha energy: 2.2476 MeV).

## Results

### Analysis of γ-emitting nuclides

Before the Cs elimination from the drain water using AMP, the solution condition and AMP amount were optimized to remove Cs so that the other elements remained (Supporting Table 1 and 2), 99.9% of Cs was removed with 0.01 g of AMP from 10 ml of 1 M HNO_3_ containing 1 μg/ml of B, Na, Mg, Al, Ca, Mn, Fe, Co, Ni, Se, Rb, Sr, Y, Zr, Mo, Ru, Rh, Ag, Sn, Sb, Te, Cs, Ba, Eu and Re, and 0.1 μg/ml of Nb. Most elements remained in the solution although the rates of remaining Rb and Ag were low and those of Zr, and Nb decreased with increasing AMP amount. The concentration of Mo in the solution was increased to dozens of μg/ml because Mo in the AMP was dissolved to the solution. Remaining rates of metal ions were also affected by HNO_3_ concentration. Increment of HNO_3_ concentration tends to enhance remaining rates of metal ions. Although Zr and Nb hardly remained in 0.1 M HNO_3_, 94% and 67% of Zr and Nb, respectively, remained in 2 M HNO_3_. Therefore, 2 M HNO_3_ condition was selected so that larger amount of Zr and Nb remain in the solution. Furthermore, the rate of remaining Sb depended on the standing time after preparation of sample solution. Although Sb existed as SbCl_3_ in the standard solution, it could be changed to Sb^5+^ in the oxidizing solution, 2 M HNO_3_, to remain in the filtrate. It seemed that a part of Sb^3+^ was captured by AMP but Sb^5+^ remained in the solution. Therefore, drain water was stood for overnight after adding acid and carrier to wait for the oxidization of Sb^3+^ to Sb^5+^.

**Figure 2:**
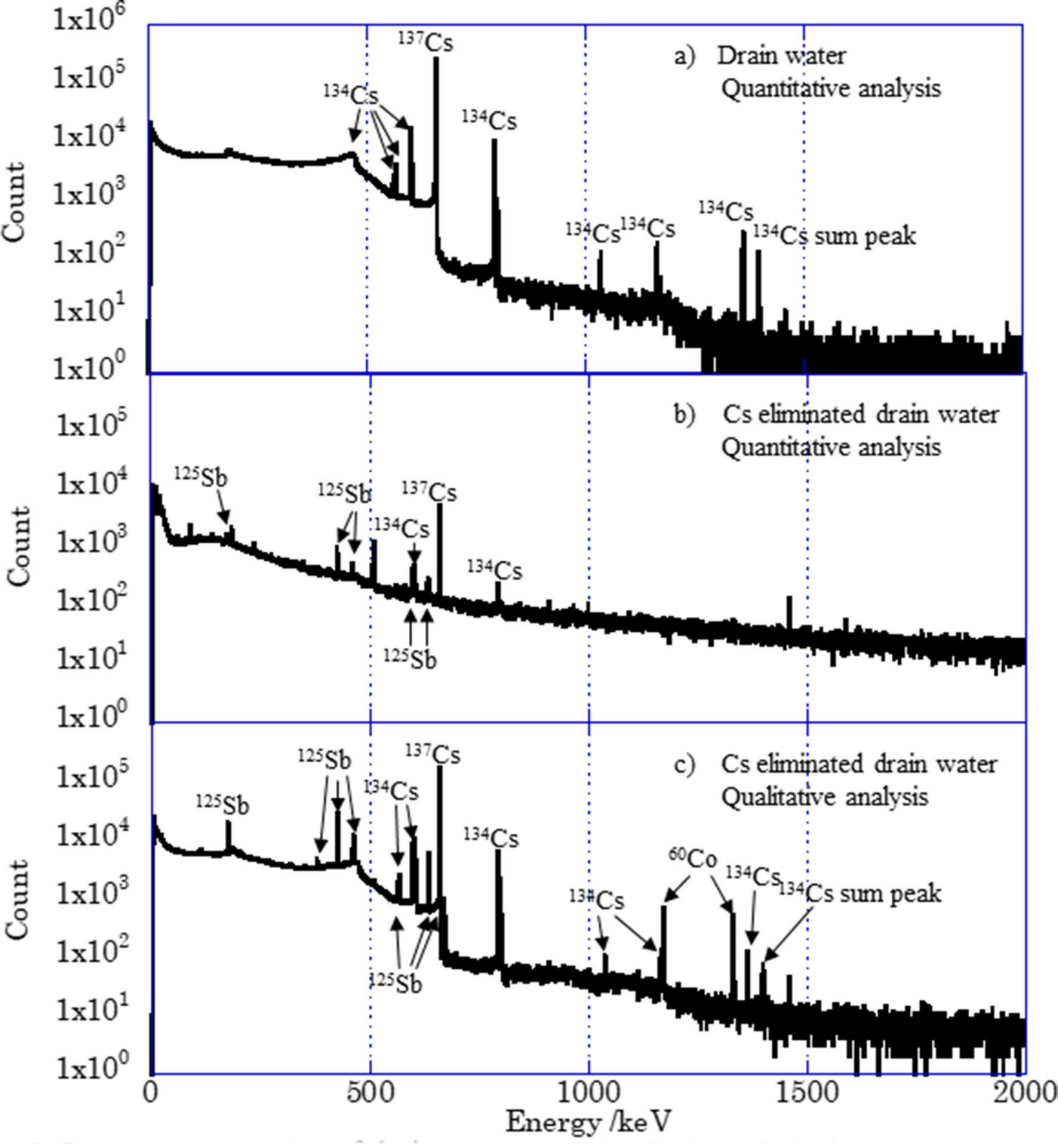
Gamma-ray spectra of drain water and Cs eliminated drain water. Sample solution (30 μl) was transfused into filter paper for quantitative analysis, (**a**) and (**b**). Sample solution in a bottle was measured for quantitative analysis, (**c**).

Figure 2 shows the γ-ray spectra for (a) quantitative analysis of drain water, (b) quantitative analysis of Cs-eliminated drain water, and (c) qualitative analysis of Cs-eliminated drain water. Only peaks of ^134^Cs and ^137^Cs were observed in spectrum (a) except those of natural background nuclides and backscattering. The concentrations of ^134^Cs and ^137^Cs were 2.4 × 10^3^ and 4.8 × 10^4^ Bq/ml, respectively. When the radioactivity was decay-corrected to March 11, 2011, they were (5.1 ± 0.02) × 10^4^ and (5.9 ± 0.007) × 10^4^ Bq/ml, respectively, and the ^134^Cs/^137^Cs radioactivity ratio was 0.86 ± 0.01.

The concentration of ^137^Cs was decreased to 63 Bq/ml for the Cs-eliminated drain water, and peaks of ^125^Sb showed up. The concentration of ^125^Sb in the drain water was 14 Bq/ml. It is reported that Sb was released by the oxidation of LWR fuel cladding by steam^38^. While not only ^125^Sb but also ^60^Co were observed for the qualitative analysis of the Cs-eliminated drain water, the concentration of ^60^Co were less than its detection limit for quantitative analysis, 0.7 Bq/ml, due to a small amount of sample, 30 μl.

### Analysis of β-emitting nuclides

**Figure 3:**
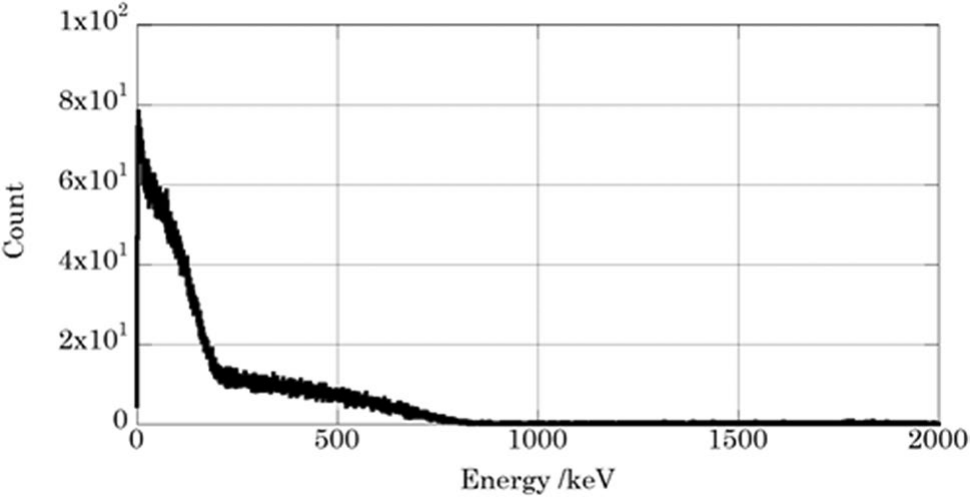
Liquid scintillation spectrum of ^90^Sr-^90^Y for the drain water after separation and reached radioactive equilibrium.

Figure 3 shows the liquid scintillation spectrum of the Sr measurement sample separated from DW-Sr sample. The spectrum consists of β-rays emitted from ^90^Sr and ^90^Y. The maximum energies were shifted to lower energy side because of chemical quenching due to HNO_3_. Recovery rate of Sr for chemical separation was (92 ± 8)%. The obtained ^90^Sr concentration was (47 ± 4) Bq/ml at the measurement time, and (55 ± 5) Bq/ml at the time point of the accident at the FDNPS.

There is no stable Tc isotope. Because Re and Tc tend to form ReO_4_^−^ and TcO_4_^−^, respectively, and show similar behavior, Re was used as a carrier of Tc. The recovery rate of Re after all chemical separation processes was 99.5%, and this result supports that the chemical separation procedure is suitable for the ^99^Tc separation. However, the concentration of ^99^Tc in the drain water was less than detection limit, 0.012 Bq/ml.

### Analysis of iodine species

In this study, organic and molecular iodine were not measured because they seemed to be vaporized during long period until sampling and analysis on September 12th, 2016, and on November 17-18th, 2020, respectively. Furthermore, it was reported that the largest fraction of the radioiodine present in BWR condensate was found to be I^−^^39^, and molecular I_2_ was hydrolyzed and disproportionated in water to produce I^−^ and IO_3_^−^^40^. Therefore, these species were considered dominant species and measured. Table 1 shows the concentrations of iodine species in the drain water. As the total inorganic iodine, 20 ± 3 and 21 ± 2 ng/ml of ^127^I and ^129^I, respectively, were obtained and the values were the same order of the reported ^129^I concentration in the accumulated water before treatment^36^. It was estimated that the amounts of ^127^I and ^129^I in Unit 1 core were 2.14 × 10^3^ and 9.44 × 10^3^ g/core, respectively^41^, whereas reported ^127^I concentrations in the rainwater collected from Fukushima prefecture were a few ng/ml level^42^. Therefore, ^127^I in the drain water is considered a mixture of Unit 1-originated ^127^I and rainwater-originated ^127^I. Although the chemical form might be changed until the analysis and measurement, most iodine was observed as I^−^ in the present time; approximately 90% of iodine was I^−^ and 10% was IO_3_^−^ in both isotopes. In case of the seawater collected at the offshore Fukushima on June 3-17th, 2011, the I^−^/IO_3_^−^ ratio for ^129^I different from that for nature-originated ^127^I was observed; the ^129^I originated from FDNPS existed mainly as I^−^ form but nature-originated ^127^I mainly as IO_3_^−^ form^43^. However, our result shows similar I^−^/IO_3_^−^ ratio for both isotopes, indicating isotope exchange reaction reached equilibrium.

**Table 1:** Concentrations and percentages for each iodine chemical species.

	^127^I	^129^I
Total iodine (ng/ml)	20 ± 3	21 ± 2
Iodide (I^−^) (ng/ml)	18 ± 3	19 ± 1
Iodate (IO_3_^−^) (ng/ml)	2.1 ± 3.4	1.9 ± 1.5
Percentage of iodide (%)	90	89
Percentage of iodate (%)	11	11

The error was estimated based on the measurement errors and their propagation.

**Table 2:** Comparison of Mo isotope ratios for natural and drain water, and Unit 1 originated one and calculated on using ORIGEN2 code.

Mass number	Natural*^1^	Mo STD after separation using TEVA resin	Drain water	Unit1 originated Mo isotopes in drain water	ORIGEN2*^2^ Unit 1
92	0.145	0.144 ± 0.034	0.125 ± 0.015	–	–
94	0.092	0.091 ± 0.028	0.080 ± 0.012	0.005 ± 0.003	–
95	0.158	0.158 ± 0.036	0.166 ± 0.017	0.210 ± 0.005	0.214
96	0.167	0.166 ± 0.037	0.147 ± 0.016	0.025 ± 0.003	0.011
97	0.096	0.096 ± 0.028	0.116 ± 0.015	0.241 ± 0.005	0.243
98	0.244	0.245 ± 0.045	0.246 ± 0.021	0.256 ± 0.007	0.250
100	0.098	0.099 ± 0.029	0.121 ± 0.015	0.264 ± 0.005	0.283

*^1^The values were reported in reference 44.

*^2^The values were calculated using the values calculated by Nishihara et al*.* in reference ^41^.

### Analysis of Mo isotopes

Stable Mo isotopes of which mass number 95, 97, 98, 100, have high fission yield, 6.497, 6.045 5.701, and 6.579%, respectively^44^. Furthermore, it was reported that Mo and Cs showed similar distribution within the corium after heating of MOX fuel at 2620℃ with hydrogen and steam condition, suggesting the formation of Cs_2_MoO_4_^45^. Therefore, Mo is an important element to consider chemical form of radiocesium.

**Figure 4:**
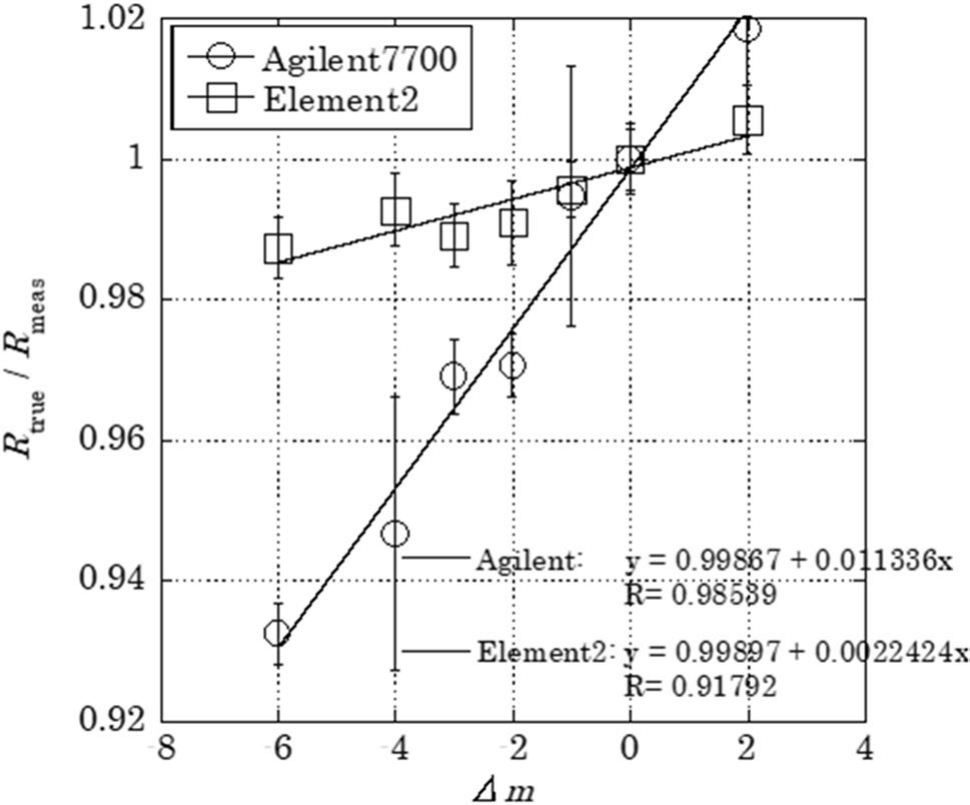
Relationship between mass difference (Δ*m*) and ration of true isotope ratio to measured value (*R*_true_/* R*_meas_).

Mass discrimination effect was evaluated to measure Mo isotope ratios precisely. Figure 4 shows the relationship between (*R*_true_/*R*_meas_) ratio and *Δ*m for the measurement of Mo standard solution using Agilent 7700 and Element 2 with relational expression. Noticeable mass discrimination effect was observed for the measurement using Agilent 7700, suggesting that the isotope ratios should be corrected by the mass discrimination factor obtained from the slope of the relational expression. On the other hand, mass discrimination effect for Element 2 was relatively small, and their dispersion was less than 1.5%. Therefore, Mo isotopes in the drain water sample was measured by Element 2 and the obtained isotope ratio was used without correction in this study.

Table 2 shows the Mo isotope ratios in the Mo standard solution and the drain water after separation using the TEVA resin. In addition, the isotope ratios of Mo generated in the Unit 1 were estimated by ORIGEN code^41,44^. Isotope ratios for the Mo standard solution after separation using TEVA resin agreed with those of natural isotope ratios, indicating isotope effect is negligible during the chemical separation, whereas Mo isotope ratios for drain water was different from the natural isotope ratios and those originated from Unit 1. It is considered that nature-originated Mo and Unit 1-originated Mo were mixed in the drain water. Molybdenum-92 in the drain water is considered nature-originated isotope because the fission yield of ^92^Mo is approximately 10 order magnitude lower than those of ^95,97,98,100^Mo^44^. Then, count rates of nature-originated Mo isotopes in the drain water were calculated from the count rate of ^92^Mo and natural isotope ratio. Next, count rates of Mo isotopes originated from Unit 1 were calculated by subtracting the calculated count rates of nature-originated Mo in the drain water from the measured count rates of Mo isotopes for the drain water. Molybdenum isotope ratios originated from Unit 1 in the drain water were calculated and listed in Table 2. They were roughly agreed with those calculated by ORIGEN code, indicating that the drain water contains Unit 1-originated Mo isotopes. This is the first measurement result of the stable Mo isotopes released from FDNPS. The concentrations of the total Unit 1-originated stable Mo and nature-originated Mo in the drain water were 0.13 and 0.82 μg/L, respectively. Considering that it is reported that sub-μg/L quantities of Mo are expected in rainwater^46^, main source of nature-originated Mo in the drain water is probably rainwater.

### Analysis of Th, U, Pu and Am

Thorium and U were not detected. The limit of detection of ICP-MS measurement for Th and U were 0.0006 and 0.0013 ng/mL, respectively, and this means that the concentrations of Th and U in the drain water were less than 0.12 and 0.25 ng/mL, respectively, because the drain water was diluted 200 times for the measurement. Also, Pu and Am were not detected in the drain water. Here, the recovery rates for Pu and Am obtained from the analytical results of ^242^Pu or ^243^Am-spiked drain water samples were 93% and 101%, respectively, and the limits of detection for ^242^Pu and ^243^Am were both 0.002 Bq.

## Discussion

### Mole ratios of determined isotopes

The measured radioactivity concentrations of target nuclides in the drain water were decay-corrected to the time point of the accident at FDNPS, March 11th, 2011, and then, the radioactivity ratios of the target nuclides to ^137^Cs were calculated (see Table 3).

**Table 3:** Radioactivity and concentrations of target isotopes in the drain water and ratios of them to ^137^Cs for the measured and calculated for Unit 1 core.

Isotopes	Concentration (Bq/ml)	Radioactivity ratio to ^137^Cs	Concentration (mol/ml)	Mole ratio to ^137^Cs	Mole ratio to ^137^Cs in Unit1 core (ORIGEN)	Ratios of drain water to Unit1 core ratios
^90^Sr	55 ± 5	9.3 × 10^−4^	1.2 × 10^−13^	8.5 × 10^−4^	0.71	1.2 × 10^−3^
^95^Mo	−	−	2.9 × 10^−13^	2.1 × 10^−3^	0.87	2.4 × 10^−3^
^96^Mo	−	−	3.4 × 10^−14^	2.4 × 10^−4^	0.04	5.6 × 10^−3^
^97^Mo	−	−	3.3 × 10^−13^	2.3 × 10^−3^	0.97	2.4 × 10^−3^
^98^Mo	−	−	3.4 × 10^−13^	2.5 × 10^−3^	0.99	2.5 × 10^−3^
^100^Mo	−	−	3.5 × 10^−13^	2.5 × 10^−3^	1.1	2.3 × 10^−3^
Total Mo	−	−	1.3 × 10^−12^	9.6 × 10^−3^	4.0	2.4 × 10^−3^
^99^Tc	N.D	−	N.D	−	7.0	−
^125^Sb	1.5 × 10^2^	2.5 × 10^−3^	3.3 × 10^−14^	2.4 × 10^−4^	0.0049	4.8 × 10^−2^
^129^I	0.14 ± 0.01	2.4 × 10^v7^	1.6 × 10^−10^	1.2	0.16	7.4
^134^Cs	(5.1 ± 0.02) × 10^4^	0.86 ± 0.01	8.0 × 10^−12^	5.7 × 10^−2^	0.065	8.9 × 10^−1^
^137^Cs	(5.9 ± 0.007) × 10^4^	1	1.4 × 10^−10^	1.0	1.0	1.0
^152^Eu	N.D	−	N.D	−	2.3 × 10^−14^	−
^154^Eu	N.D	−	N.D	−	0.012	−
^238^U	N.D	−	N.D	−	597	−
^241^Am	N.D	−	N.D	−	0.040	−
^239^Pu	N.D	−	N.D	−	2.8	−

※The concentrations were decay-corrected to the time of the accident, March 11, 2011.

TEPCO and some researchers reported radioactivity concentrations of ^134^Cs and ^137^Cs in stagnant water in the FDNPS^47–49^. The ^134^Cs/^137^Cs radioactivity ratios for stagnant water of Unit 1 decay-corrected to March 11th, 2011 were varied from 0.89 to 0.97 and the averages reported by Komori et al. and Nishihara et al. were 0.93 ± 0.03 and 0.92 ± 0.02, respectively. Furthermore, the radioactivity ratio reported by Komori et al. was 0.91 ± 0.2 for the stagnant water sampled before October, 22th 2011 to avoid mixture of stagnant water from Unit 2 due to transportation of stagnant water from Unit 1 to Unit 2. Our value, 0.86, is slightly smaller than these reported values. It is reported that neutron spectrum and flux are not uniform throughout a reactor core^50,51^. In consequence, variety occurs in the Cs isotopic composition as a function of location within the core: the upper side had smaller ^134^Cs/^137^Cs radioactivity ratio. In addition, Snow et al. suggested that (i) when power excursion and/or loss of coolant happened rapidly, Cs isotope ratio would be similar to total core ratio, ii) when coolant loss happened gradually over the course of minutes to hours, radiocesium component would be changed^51^. Compare to the variety of Cs isotope composition in reactor core, the difference in the obtained ^134^Cs/^137^Cs radioactivity ratios is small and close to the ratio in the Unit 1 core. Therefore, power excursion and/or loss of coolant is possibly happened rapidly.

The measured ^134^Cs/^137^Cs radioactivity ratio for stagnant water of Unit 1 was clearly smaller than those for the stagnant water of Units 2 and 3 (1.00 ± 0.03), and clearly smaller ^134^Cs/^137^Cs radioactivity ratio was observed at north-northwest site near FDNPS and Oshika Peninsula^27–29,48^. Although released amounts of radionuclides by vent operation of the Unit 1 was estimated smaller than those by explosion, the ^134^Cs/^137^Cs radioactivity ratio could become an index for the source term analysis.

The ^90^Sr/^137^Cs radioactivity ratio in the drain water on March 11th, 2011 is calculated to be 9.3 × 10^−4^. Nishihara et al. and Asai et al. reported ^90^Sr radioactivity in the stagnant water of Unit 1 collected on 24th and 27th of March 2011^49,52^. The calculated ^90^Sr/^137^Cs radioactivity ratio on 11th of March from the reported values was 1.3 × 10^−4^, which is one order magnitude lower than that of the drain water. On the other hand, the ^90^Sr/^137^Cs radioactivity ratio for rubble collected at the 1st floor of Unit1 on October 2013 and at just outside of Unit 1 on June 25th, 2012 and July 26th and 27th, 2012 was 1.5 × 10^−3^–5.0 × 10^−3^ and 1.4 × 10^−3^–5.6 × 10^−3^, respectively^53,54^, and these values were close to our value. In addition, it is reported that ^90^Sr concentration in the accumulated water increased gradually over time and reached almost comparable to ^137^Cs concentration in early 2012^55^. Since transportation process of ^90^Sr during accident and after accident would be different, the sampling location and time should be noted for consideration. This complicated transportation processes of Sr may cause wide variation of ^90^Sr/^137^Cs radioactivity ratio for environmental samples^56–59^.

In order to discuss the chemical form of radiocesium, mole ratio is important rather than radioactivity ratio. Therefore, radioactivity concentrations were converted to mole concentrations, and the mole concentrations of the target nuclides were divided by the mole concentration of ^137^Cs to obtain their mole ratios to ^137^Cs. It is considered that the radionuclides in the drain water were originated from vent operation of the Unit 1. Therefore, the amounts of radionuclides in the Unit 1 core were considered as original inventory. The mole ratios of the target nuclides to ^137^Cs in the Unit 1 core were calculated by using the reported values of ORIGEN calculation and listed in Table 3. Furthermore, the mole ratios to ^137^Cs obtained from the measurement were divided by the mole ratios to ^137^Cs obtained from ORIGEN calculation (hereafter referred to as the ratio of drain water to Unit 1 core ratios). These results are summarized in Table 3. The order of ratios of the drain water to Unit 1 core ratios was ^129^I > ^137^Cs≈^134^Cs > ^125^Sb > Mo > ^90^Sr. It is reported that Mo release increased under oxidizing conditions with sufficient water vapor inside reactor core through the formation of volatile species^60^. However, the amount of Mo was quite low compared to that of Cs in the case of this study, suggesting that the formation of Cs_2_MoO_4_ had been suppressed under steam-starved conditions.

Based on the ORIGEN calculation, isotope mole ratio of ^129^I to the total I and ^137^Cs to the total Cs in Unit 1 core were calculated to be 78.69% and 40.27%, respectively, at the accident time^41^ From the isotope mole ratio and measured concentration of ^129^I and ^137^Cs in the drain water, the concentration of the total I and the total Cs were calculated to be 2.1 × 10^−10^ and 3.4 × 10^−10^ mol/ml, respectively, and the ratio of the total I to the total Cs was 0.6 in the drain water. Compared to the ratio of the total I to the total Cs in the Unit1 core, 0.08, the ratio in the drain water was approximately one order magnitude larger. This trend implies that major chemical form of I was molecular I rather than CsI.

## Conclusion

In order to study the behavior of radionuclides released during venting of Unit 1 at FDNPS, radiochemical analysis was conducted for the drain water sampled from the drain pit of the exhaust stack shared between Units 1 and 2.

Although γ-ray spectra of the drain water showed only peaks related to ^134^Cs and ^137^Cs, ^60^Co and ^125^Sb were detected after elimination of radiocesium using ammonium phosphomolybdate. The decay-corrected ^134^Cs/^137^Cs radioactivity ratio to March 11th, 2011 was 0.86.

In this study, Unit 1-originated stable Mo isotopes were clearly detected. Their amounts were quite low compared to Cs, suggesting that the formation of Cs_2_MoO_4_ was suppressed under the accident condition.

Inorganic iodine chemical forms in samples collected at the FDNPS were analyzed, and it was found that approximately 90% of iodine existed as I^−^ and 10% as IO_3_^−^ in the samples. Although it is a big subject to extrapolate the present result to accident time, the information related to the iodine chemistry was obtained for the first time.

The ratios of the mole concentration ratios of nuclides to ^137^Cs in the reactor core to those in the drain water was calculated: The order was ^129^I > ^137^Cs≈^134^Cs > ^125^Sb > Mo > ^90^Sr. The larger ratio for ^129^I implies that iodine was released as molecular iodine rather than CsI.

The results and information obtained from this study could be used as source terms to analyze contamination of environment, and would be applied for the accident analysis of Unit 1 at FDNPS to discuss chemical form of released radionuclides and reactor condition of the accident time.

## Supplementary Information

Supplementary Information.

## Abbreviations

FDNPS: Fukushima Daiichi Nuclear, Power Station
AMP: Ammonium phosphomolybdate
