# Supplementary material for: Radiochemical analysis of the drain water sampled at the exhaust stack shared by Units 1 and 2 of the Fukushima Daiichi Nuclear Power Station
Source: Sci Rep. 2022 Feb 8;12:2086. doi: 10.1038/s41598-022-05924-2 (PMC8825831; doi:10.1038/s41598-022-05924-2)
Supplement: Supplementary file 1 — Supplementary Information. [file 41598_2022_5924_MOESM1_ESM.docx]

**Supporting Table 1.** Remain rates of elements in filtrate after Cs elimination using various amount of AMP

|  | Remain rate /% | | | | | | |
| --- | --- | --- | --- | --- | --- | --- | --- |
| Element | Amount of AMP | | | | | | |
|  | 0.001g | 0.005g | 0.01g | 0.02g | 0.05g | 0.1g | 0.5g |
| B | 101 | 99 | 99 | 98 | 95 | 99 | 99 |
| Na | 103 | 106 | 106 | 103 | 100 | 101 | 108 |
| Mg | 96 | 94 | 96 | 98 | 98 | 102 | 103 |
| Al | 100 | 97 | 97 | 96 | 100 | 206 | 222 |
| Ca | 108 | 113 | 107 | 104 | 102 | 103 | 105 |
| Mn | 98 | 97 | 99 | 99 | 96 | 103 | 100 |
| Fe | 100 | 101 | 100 | 99 | 99 | 101 | 97 |
| Co | 97 | 97 | 99 | 99 | 97 | 101 | 99 |
| Ni | 98 | 97 | 99 | 98 | 97 | 100 | 100 |
| Se | 93 | 99 | 98 | 101 | 93 | 103 | 91 |
| Rb | 26 | 5.0 | 3.7 | 2.9 | 2.5 | 1.7 | 1.2 |
| Sr | 99 | 98 | 101 | 101 | 98 | 100 | 99 |
| Y | 98 | 97 | 98 | 97 | 93 | 95 | 86 |
| Zr | 95 | 92 | 86 | 72 | 49 | 40 | 14 |
| Nb | 82 | 75 | 78 | 53 | 20 | 12 | 4.5 |
| Mo | 2320 | 4124 | 5058 | 3485 | 5009 | 7199 | 12125 |
| Ru | 97 | 98 | 95 | 94 | 94 | 97 | 93 |
| Rh | 99 | 99 | 99 | 99 | 96 | 100 | 98 |
| Ag | 6.8 | 6.9 | 44 | 43 | 18 | 20 | 19 |
| Sn | 97 | 97 | 101 | 100 | 95 | 97 | 96 |
| Sb | 94 | 79 | 101 | 101 | 98 | 102 | 99 |
| Te | 98 | 97 | 103 | 104 | 98 | 100.1 | 100 |
| Cs | 1.1 | 0.2 | 0.1 | 0.1 | 0.1 | 0.1 | 0.1 |
| Ba | 99 | 99 | 101 | 101 | 97 | 98 | 98 |
| Eu | 98 | 98 | 99 | 98 | 95 | 98 | 92 |
| Re | 96 | 96 | 97 | 98 | 95 | 99 | 95 |

10 ml of MES containing 1 μg/ml of elements except Nb and 0.1 μg/ml of Nb in 1 M HNO_3_ was used.

**Supporting Table 2.** Remain rates of elements in filtrate after Cs elimination using various acid conditions

| Element | HNO_3_ | | | |  | HCl | | | |
| --- | --- | --- | --- | --- | --- | --- | --- | --- | --- |
|  | 0.1 M | 0.5 M | 1 M | 2 M | H_2_O | 0.1 M | 0.5 M | 1 M | 2 M |
| B | 96 | 105 | 98 | 101 | 97 | 98 | 112 | 104 | 99 |
| Na | 100 | 106 | 103 | 101 | 96 | 91 | 104 | 105 | 90 |
| Mg | 93 | 103 | 98 | 102 | 53 | 91 | 103 | 104 | 104 |
| Al | 32 | 96 | 96 | 97 | 26 | 36 | 103 | 101 | 99 |
| Ca | 94 | 105 | 104 | 106 | 50 | 93 | 106 | 105 | 104 |
| Mn | 92 | 99 | 98 | 100 | 51 | 92 | 99 | 99 | 99 |
| Fe | 19 | 94 | 99 | 102 | 16 | 43 | 97 | 101 | 104 |
| Co | 93 | 98 | 99 | 100 | 48 | 93 | 99 | 99 | 99 |
| Ni | 91 | 99 | 98 | 99 | 46 | 92 | 98 | 100 | 98 |
| Se | 92 | 98 | 101 | 97 | 66 | 97 | 100 | 99 | 101 |
| Rb | 2 | 3 | 3 | 4 | 2 | 2 | 2 | 2 | 3 |
| Sr | 92 | 99 | 101 | 100 | 44 | 91 | 99 | 99 | 100 |
| Y | 10 | 79 | 97 | 99 | 0 | 9 | 80 | 98 | 99 |
| Zr | 5 | 35 | 72 | 94 | 2 | 5 | 33 | 71 | 95 |
| Nb | 4 | 17 | 53 | 67 | 0 | 3 | 27 | 51 | 65 |
| Ru | 53 | 93 | 94 | 98 | 16 | 93 | 100 | 101 | 99 |
| Rh | 90 | 98 | 99 | 99 | 67 | 91 | 99 | 100 | 100 |
| Ag | 4 | 5 | 43 | 12 | 4 | 30 | 96 | 98 | 98 |
| Sn | 46 | 87 | 100 | 101 | 2 | 55 | 90 | 94 | 89 |
| Sb | 90 | 99 | 101 | 100 | 32 | 66 | 65 | 85 | 99 |
| Te | 101 | 102 | 104 | 103 | 99 | 101 | 101 | 100 | 100 |
| Cs | 0.1 | 0.1 | 0.1 | 0.1 | 0.1 | 0.1 | 0.1 | 0.1 | 0.1 |
| Ba | 90 | 100 | 101 | 103 | 33 | 86 | 98 | 101 | 101 |
| Eu | 9 | 80 | 98 | 102 | 0 | 8 | 76 | 97 | 100 |
| Re | 92 | 94 | 98 | 100 | 78 | 81 | 85 | 87 | 90 |

10 ml of MES containing 1 μg/ml of elements except Nb and 0.1 μg/ml of Nb in various acid solution was used. The amount of AMP was 0.02 g.

**Supporting Table 3.** Measured count rate for standard solution and drain water after separation using TEVA resin and calculated count rate for natural Mo and Unit1 originated Mo

| Mass number | Net cps | | Calculated cps | |
| --- | --- | --- | --- | --- |
|  | Mo STD after separation using TEVA resin | Drain water | Nature-originated Mo isotopes in drain water | Unit 1 originated Mo isotopes in drain water |
| 92 | 152607±1237 | 147706±292 | 147706±292 | - |
| 94 | 96622±952 | 93791±314 | 93015±184 | 776±364 |
| 95 | 166673±1167 | 195124±447 | 161023±318 | 34101±548 |
| 96 | 175722±1394 | 173449±357 | 169460±334 | 3989±490 |
| 97 | 101679±835 | 136707±306 | 97590±193 | 39117±362 |
| 98 | 259450±2101 | 289570±568 | 247938±489 | 41632±750 |
| 100 | 105046±1159 | 142718±64 | 99826±197 | 42892±207 |
